# Natural Killer Cells: Tumor Surveillance and Signaling

**DOI:** 10.3390/cancers12040952

**Published:** 2020-04-11

**Authors:** Lizeth G. Meza Guzman, Narelle Keating, Sandra E. Nicholson

**Affiliations:** 1 The Walter and Eliza Hall Institute of Medical Research, Parkville, VIC 3052, Australia; keating.n@wehi.edu.au; 2 Department of Medical Biology, The University of Melbourne, Parkville, VIC 3010, Australia

**Keywords:** natural killer cells, NK cells, immune surveillance, signaling, inhibitory receptors, activating receptors

## Abstract

Natural killer (NK) cells play a pivotal role in cancer immunotherapy due to their innate ability to detect and kill tumorigenic cells. The decision to kill is determined by the expression of a myriad of activating and inhibitory receptors on the NK cell surface. Cell-to-cell engagement results in either self-tolerance or a cytotoxic response, governed by a fine balance between the signaling cascades downstream of the activating and inhibitory receptors. To evade a cytotoxic immune response, tumor cells can modulate the surface expression of receptor ligands and additionally, alter the conditions in the tumor microenvironment (TME), tilting the scales toward a suppressed cytotoxic NK response. To fully harness the killing power of NK cells for clinical benefit, we need to understand what defines the threshold for activation and what is required to break tolerance. This review will focus on the intracellular signaling pathways activated or suppressed in NK cells and the roles signaling intermediates play during an NK cytotoxic response.

## 1. Introduction

Natural Killer (NK) cells are bone marrow–derived innate lymphocytes that are found in most organs, with the largest population of NK cells residing in the blood [1]. NK cells are large granular lymphocytes that were initially defined by their ability to kill tumor cells without prior sensitization [2,3]. The role of NK cells has since been expanded to include the elimination of virally infected cells and secretion of cytokines that mediate crosstalk and regulation of other immune cells [4].

Following their discovery in the 1970s, immunologists have been fascinated by the ability of NK cells to detect and kill tumorigenic or virally-infected cells, whilst tolerating healthy self-tissue [5,6,7]. However, it wasn’t until the early 1990s that scientists started to explore the mechanisms by which NK cells distinguished “self” from “non-self,” an area of research instigated by Klas Kärre’s exposition of the “missing self” theory [8]. Karre hypothesized that NK cells could recognize loss or reduction in surface expression of major histocompatibility complex (MHC) class I proteins (human leukocyte antigens (HLA) class I in humans), triggering recognition as non-self. This hypothesis was based on earlier studies by Strokus et al. [9,10] that described protection of susceptible cells with experimentally expressed MHC-I. The ‘missing-self’ theory was further validated by Karlhofer et al. [11], who showed that the murine Lymphocyte Ag 49A (Ly49A) receptor recognized and discriminated between different MHC-I molecules, with tumor cells from H2d and H2K backgrounds resistant to killing by Ly49A expressing NK cells [11,12]. Soon after, Moretta et al. [13] discovered the first human inhibitory NK receptor Killer cell immunoglobulin-like receptor 2DL1 (KIR2DL). Inhibitory receptors are now known to not only sense reduction in expression of MHC class I proteins, but also recognize non-MHC-I molecules, such as glycans and collagen, which are crucial for NK cell discrimination of self [14]. Upon engagement of cognate ligands, the various NK cell receptors send activating and inhibitory signals, which collectively determine NK cell action.

**Figure 1:**
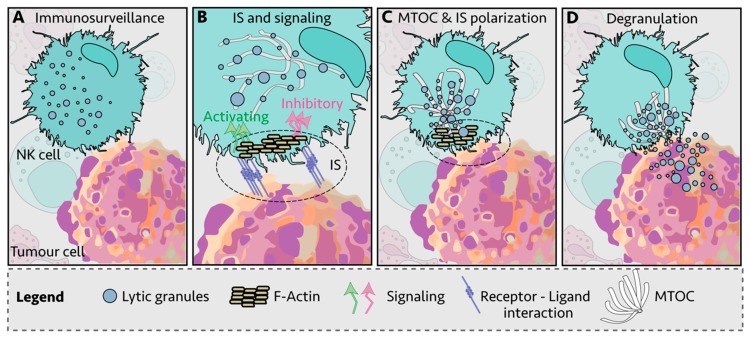
Immunological synapse (IS) of Natural Killer (NK) cell and target cell. (**A**) NK cells engage other cells via integrins and adhesion molecules, which create the immunological synapse (IS)—the subsequent process between engagement and killing or tolerance can be broken down into four steps. (**B**) First, filamentous actin (F-actin) is recruited to the IS. Inside-out signaling reinforces IS interactions and activating and inhibitory surface receptors cluster at the IS. (**C**) Second, NK lytic granules move along microtubules by dynein-dynactin motor proteins toward the microtubule-organizing center (MTOC). (**D**) Third, the polarized lytic granules and MTOC travel in an ATP-dependent manner through the actin mesh via myosin IIA to dock at the IS, and finally, the lytic granules fuse with the membrane and release the lytic contents into the target cells, a process also known as degranulation. The NK cell then detaches and moves on to the next target.

NK cells kill infected and transformed cells via a variety of mechanisms, including the delivery of lytic granules loaded with proteases and pore-forming proteins such as granzymes and perforin, release of cytokines such as tumor necrosis factor alpha (TNFα) and interferon gamma (IFNγ), upregulation of FASL and TNF-related apoptosis-inducing ligand (TRAIL) and by antibody-dependent cellular cytotoxicity (ADCC) [15,16,17,18,19]. There are multiple steps between NK cell: target cell engagement and cell killing, with receptor-ligand interactions thought to be the initiating step in the formation of an immunological synapse (IS) (Figure 1A,B) [20,21]. This is followed by recruitment of filamentous actin (F-actin) to the IS (Figure 1B) and polarization of the lytic granules and the microtubule-organizing center (MTOC) toward the IS (Figure 1C). Then, the granules dock at the synapse and are ready for the final step: granule-membrane fusion and release of the cytotoxic contents at the center of the IS (Figure 1D) [22,23]. NK cell signaling and killing is considered to be localized to the IS [24], with each NK cell thought to reach exhaustion after killing four to seven target cells [25].

NK cells represent approximately 10% of the circulating lymphocyte population and thus predominantly control hematologic malignancies and tumor metastasis, rather than solid tumors [26]. This is reflected by only minor NK cell infiltration in nascent and fully developed tumor microenvironments, such as in colorectal cancer “encapsulated” by tissue barriers [27,28]. Importantly, the presence of circulating NK cells is inversely correlated with metastatic burden in patients suffering from different carcinomas [29,30,31], gastrointestinal sarcoma (GIST) [32], melanoma [33], and breast cancer [34] and suggests that enhancing NK infiltration into tumors and/or activity would have clinical benefit. Early clinical trials for non-Hodgkin’s lymphoma and chronic lymphocytic leukemia (CLL) have shown positive patient responses following adoptive transfer of allogeneic chimeric antigen receptor (CAR)-NK cells, without significant toxicities [35]. This recent advance by Liu et al. [35] underscores the clinical utility of NK cell–based therapies.

The remainder of this review highlights the importance of individual NK receptors and our current understanding of how they function. Understanding the interplay between NK cell, tumor, and TME has the potential to not only lead to new cancer therapies, but inform which patients are likely to respond to receptor-focused interventions.

## 2. Receptor Mediated Inhibition of NK Cells: Inhibitory Receptors

**Figure 2:**
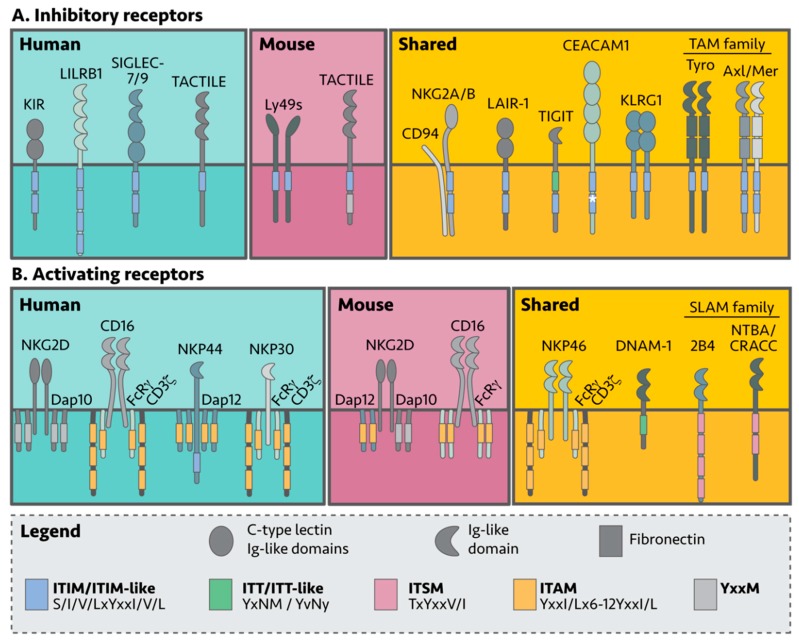
NK cell surface receptors involved in tumor recognition. NK cells express a myriad of inhibitory and activating receptors designed to recognize healthy or aberrant (non-healthy) cells. (**A**) Inhibitory receptors dampen activating NK cell signals via cytoplasmic tyrosine motifs in their cytoplasmic tails, regulating NK cell effector function. (**B**) In contrast, most activating receptors signal through cytoplasmic adaptor proteins. Although many of the receptors are expressed by both mouse and human NK cells (shared), some are exclusive.

NK cell self-tolerance in humans and mice is mostly mediated by inhibitory receptors that recognize either MHC-I complexes or non-MHC-I surface molecules [36]. In general, inhibitory receptors belong to receptor families comprised of both activating and inhibitory members, and signal through a cytoplasmic signaling tail containing an immunoreceptor tyrosine-based inhibitory motif (ITIM) (Table 1, Figure 2) [37]. NK killing of target cells can be viewed as an internal decision-making process using a “pros and cons” list; when activating signals outweigh inhibitory signals, the NK cell becomes cytotoxic. Interestingly, NK cells lacking inhibitory receptors are unable to become cytotoxic, thus acquiring functional maturation is also dependent on inhibitory signals. This requirement is commonly referred to as NK cell “education” [38].

### 2.1. MHC-I Recognizing Receptors

MHC-I complexes can be divided into classical (MHC-Ia) and non-classical (MHC-Ib) complexes [39]. The up-regulation of particular inhibitory receptors leads to NK cell education by either classical MHC-Ia-dependent (licensing), non-classical MHC-Ib-dependent recognition, or MHC-independent recognition [40].

Inhibitory MHC receptors are divided into the following three categories: the KIR and Leukocyte Immunoglobulin-Like Receptor (LILR, LIR, ILT, CD85) family in humans; the Ly49 family, also known as the Killer Cell Lectin-like Receptor subfamily A (klra) in mice; and lastly, the CD94/NKG2 receptor family, found in both mouse and human (Figure 2A). KIRs and Ly49s are so-called functional homologues as they bind MHC-I molecules yet are different at the protein and genetic sequence level [41,42]. Despite these differences, they both appear to have arisen from multiple duplication events, making KIRs and Ly49s highly polymorphic.

Inhibitory (i)KIRs are composed of a long (L) intracellular ITIM-containing region, a transmembrane domain, and an extracellular region with either two or three C2-type immunoglobulin (Ig)-like domains (2/3 D) [43,44]. iKIR family members are named based on their domain architecture, thus iKIRs can be either KIR2DL1-3,5 or KIR3DL1-5. Human NK cells express up to six iKIRs that are able to differentiate between HLA allotypes, as well as recognize different moieties on HLA molecules (Table 2). For example, KIR3DL2 recognizes HLA-A3 and -A11 allotypes, KIR3DL1 recognizes HLA-B allotypes containing a Bw4 epitope, and KIR2DL1-3 recognizes HLA-C [45,46,47]. In combination, these KIRs cover all HLA-isoforms.

The LILR family includes many inhibitory receptors, but only LILRB1 is expressed on NK cells and is capable of recognizing both classical and non-classical HLA-I molecules [48,49]. LILRB1 has four Ig-like domains in the extracellular domain and four cytoplasmic ITIMs [50,51]. Unlike other inhibitory receptors, LILRB1 is mostly known as a receptor for viral infection and until recently, was thought to have little or no effect in tumor immunity. However, given that different breast and colorectal patient cancers upregulate the LILRB1 ligand HLA-G as a means of evading NK targeting and killing, LILRB1 is likely to be important in tumor immunity [52,53,54,55].

The Ly49 receptor family contains 23 different members that are mostly encoded by a highly polymorphic Ly49 gene cluster located on chromosome 6 [90]. Although most members are expressed on NK cells, not all are expressed in all mice strains. NOD and 129 mice express most Ly49 members (15 and 13), with C57BL/6 (11) and BALB/c mice expressing relatively few members (8) [56,58,91]. Inhibitory Ly49 receptors (iLy49s) function as homodimers; each subunit is composed of a single C-type lectin domain (CTLD), an extracellular stalk region, a transmembrane region and a C-terminal cytoplasmic tail containing an ITIM. iLy49s bind both classical and non-classical MHC-I molecules through a binding site created by homodimerization of the two single CTLDs [56,57,58]. Although iLy49 binding requires MHC-I loaded with peptide, binding occurs in a peptide-independent manner, except for Ly49C, which has been shown to confer protection by binding H2-Kb haplotype loaded with specific peptides (from ovalbumin, vesicular stomatitis virus, and elongation factor, but not peptide from Sendai virus) [57,92]. Of the iLy49s present on murine NK cells, only Ly49A and Ly49C bind non-classical MHC-I ligands H2-M3 and H2-Q10, respectively [77,78,93].

The CD94:NKG2 receptor complex consists of a heterodimer between CD94 (also known as killer cell lectin-like receptor subfamily D; KLRD1) and a member of the greater NKG2 family and contributes to non-classical MHC-I-dependent education [59]. When the complex contains NKG2A or NKG2B (a splice variant of NKG2A), the receptor complex is inhibitory; however, when CD94 is complexed with other NKG2 family members it can generate an activating signal [94]. The inhibitory complex signals through two ITIMs present in the cytoplasmic tail of NKG2A and B [95,96]. NKG2A and B are the only two inhibitory NKG2 family members and recognize the same non-classical MHC-1b ligand: HLA-E in humans or its homologue Qa-1 in mice [59,60]. NKG2A and B expressing NK cells degranulate better and produce more IFNγ in the absence of HLA-E or Qa-1 [97,98] and are better killers compared to NKG2A and B negative NK cells which are considered to be uneducated. Although the activating receptor subunit NKG2C binds the same non-classical MHC-1b ligand as iNKG2A and B, the iNKG2A and B receptors display considerably higher ligand affinity, explaining why the NKG2-HLA-E/Qa-1 interaction is mostly considered relevant for maintaining self-tolerance and not activation [99].

### 2.2. Non-MHC-I Recognizing Receptors

NK cells also express various inhibitory receptors that recognize non-MHC molecules on healthy cells. These include T cell immunoglobulin and ITIM domain (TIGIT); carcinoembryonic Ag cell adhesion molecule 1 (CEACAM1); soluble leukocyte-associated Ig-like receptor-1 (LAIR-1); Killer cell lectin-like receptor G1 (KLRG1) and NKR-P1(A/B); sialic acid-binding immunoglobulin-like lectin (Siglec); and the Tyro3, Axl, and MerTK (TAM) receptors [100,101]. Like the KIRs, the TIGIT, TACTILE (CD96), LAIR-1, and CEACAM1 receptors are members of the Ig-like superfamily and share a similar domain architecture comprised of an extracellular region containing the Ig-like domains, a transmembrane domain and a cytoplasmic signaling tail (Figure 2A).

TIGIT and TACTILE bind to the poliovirus receptor (CD155, PVR) and nectin-2 (CD112, PVRL2), ligands which are normally expressed by antigen presenting cells and T cells [82,83]. However, CD155 and CD112 are also frequently expressed by tumorigenic cells, presumably as an immune escape mechanism. NK cells try to counter this evasion tactic by expressing DNAX accessory molecule-1 (DNAM-1; also known as CD226), which competes with TIGIT and TACTILE for binding to CD155 and CD112 [102]. TIGIT has the highest affinity for the ligands, followed by TACTILE, with DNAM-1 having the lowest affinity, suggesting that NK expression of DNAM-1 may not be a particularly effective counter-tactic [103,104]. The cytoplasmic signaling tails of human and murine TIGIT contain an ITIM and ITT motif, whereas murine TACTILE has only an ITIM motif, and human TACTILE has an additional YxxM activating motif (Table 3) [105]. TIGIT is the most well understood and blocking TIGIT gives rise to a potent NK anti-tumor effect, which is attributed to both loss of NK inhibition and enhanced activation. Recently, Zhang et al. [83] showed that TIGIT blockade appeared to reverse NK cell exhaustion, as well as enhance NK mediated anti-tumor responses in an experimental colon cancer model, both alone and in combination with anti-PD1 (programmed cell death protein 1) and anti-PD-L1 (programmed death-ligand 1).

PD-1 and PD-L1 are well-known T cell checkpoints commonly targeted in cancer immunotherapy [106]. PD-1 recognizes two ligands, PD-L1 (B7-H1) and PD-L2 (programmed death-ligand 2), but only PD-L1 is constitutively expressed on hematopoietic and non-hematopoietic cells, explaining why intervention has focused on the PD-1:PD-L1 interaction [107,108]. PD-1 and PD-L1 contain a similar domain architecture encompassing an Ig-like extracellular binding domain, a transmembrane region, and a cytoplasmic tail [109,110]. The cytoplasmic tail of PD-1 contains an ITIM and ITSM motif, while the cytoplasmic tail of PD-L1 is short, lacks the motifs and does not have a known function.

In recent years, multiple studies have investigated a role for these checkpoints in NK cells, with no clear consensus reached in the field. Some studies suggest that PD-1 is expressed solely on activated NK cells [111], while others have identified PD-1 expression as a marker for dysfunctional or exhausted NK cells [112,113,114]. Most recently, Judge et al. [115] compared PD-1 expression on mouse, human- and canine, T cells and NK cells under various conditions in vitro and investigated intratumoral NK cells in sarcoma, colon cancer, and head and neck squamous carcinoma, concluding that PD-1 was not significantly expressed on mouse or human NK cells. However, there may still be an argument for combining anti-PD-1 therapy with other checkpoint blockers to rescue exhausted NK cells [114].

T cell immunoglobulin and mucin domain-containing protein 3 (TIM-3) and Lymphocyte-activation gene 3 (LAG-3) are newly established T cell checkpoints and targets for cancer therapy, with studies suggesting an emerging role for these proteins in NK cells [116]. TIM-3 recognizes galectin-9, phospholipid phosphatidylserine (PtdSer), alarmin high mobility group box 1 (HMGB1) and carcinoembryonic antigen-related cell adhesion molecule 1 (CEACAM-1) via its Ig-like extracellular domain [117,118,119,120]. Although the intracellular tail of TIM-3 contains five tyrosines that are conserved between humans and mice and are potential phosphorylation sites, they don’t conform to known binding motifs [121]. TIM-3 is expressed by NK cells and is reported to not only inhibit NK cell cytotoxicity [122], but also mediate IFNγ production [123]. So et al. were unable to reproduce these specific findings and argued that TIM-3 could be used as a marker for end-stage NK cell activation, with TIM-3 blockade likely to affect NK cell activation [124].

LAG-3 binds to MHC-II on APCs via four Ig-like domains in the extracellular region and signals via a “KIELE” motif present in the cytoplasmic tail [125,126,127]. Although NK cells express LAG-3, studies exploring its biological relevance are still limited. LAG-3 deficiency in mice resulted in decreased NK cell cytotoxicity [128], while anti-LAG-3 blocking antibodies had no impact on human NK cells [129], suggesting a species-specific role. Regardless, LAG-3 in NK cells requires further investigation before being considered as a candidate for immune checkpoint therapy.

LAIR1 binds to all collagens and is thought to aid in NK cell discrimination of damaged tissue, where the collagen-rich extracellular matrix of cells has been reduced [130]. Tumor cells have been observed to up-regulate localized collagen expression as a means of forming adhesive structures in the TME. Coincidentally, this also hides tumor cells from LAIR-1-expressing immune cells such as NK cells, with LAIR-1 inhibitory signals correlating with dampened in NK cytotoxicity [85].

Siglec-7 and 9 receptors recognize sialylated glycans [131,132], which are found on glycoproteins and glycolipids on the outer membrane of mammalian cells and are believed to act as a signal of self [133]. An abnormally high sialic acid coat (hypersialylation) is a hallmark of many tumors and may facilitate evasion of Siglec-expressing NK cells [134,135]. Consistent with this, several in vitro studies using blocking antibodies or chemical de-sialylation of proteins on the surface of tumor cells have demonstrated enhanced NK-mediated anti-tumor responses [136,137,138]. Although a murine orthologue for Siglec-9 exists (Siglec-E), it is not expressed on NK cells, so there is no role for Siglec 9 NK responses in mice to date. 

The CEACAM family is composed of over 10 members that have multiple roles in differentiation, proliferation and signaling. CEACAM1 is the only family member expressed on NK cells and has approximately 12 isoforms with varying extracellular and intracellular domains. The CEACAM1-L isoform predominates and contains three to four extracellular Ig-like domains and two cytoplasmic ITIMs in humans, or one ITIM and one ITSM in rodents [143]. NK CEACAM1 binds other CEACAM molecules present on nearby cells, including tumor cells; this interaction inhibits NKG2D-mediated tumor control [84,149].

The C-type lectin receptor superfamily includes inhibitory members KLRG1 on both human and mice, NKRP1A in humans, and NKRP1B and gp49B1 in mice. KLRG1 is mostly known for its role in NK cell maturation, development and maintenance of peripheral homeostasis [150]. Recently, a new role for KLRG1 has emerged as an inhibitory receptor in the context of tumor control. The KLRG1 ligands E- and N-cadherin were found to be upregulated in tumor samples from patients suffering from melanoma, prostate, breast or colorectal cancer [86]. Furthermore, blocking KLGR1 in mice showed enhanced tumor control compared to controls, with even better protection observed when used in combination with anti-PD1 [86]. Little research has been performed to understand the inhibitory role of KLRG1 in NK cells and this latter study highlights the therapeutic potential of anti-NKLRG1 antibodies.

The TAMs are a relatively new family of NK inhibitory receptors [88,151,152]. TAMs contain an extracellular region composed of two Ig-like domains followed by two fibronectin type II domains, a transmembrane region and an intracellular region containing a tyrosine kinase domain, an ITIM and ITIM-like motif. TAMs are classified as receptor tyrosine kinases (RTKs) and bind via their extracellular domain to growth arrest-specific gene 6 (Gas6) and Protein S (Pros1). TAM inhibition in the mouse B16F10 melanoma model has shown promising results and mechanistically, TAM receptors are proposed to act via inhibition of NKG2D signaling [88]. TAM association with Casitas B lineage lymphoma b (Cbl-b) has recently uncovered a new inhibitory signaling pathway in NK cells [88,153] (discussed in more detail in a later section; Figure 2A).

## 3. Current Therapies Harnessing the Power of Inhibitory NK Receptors

The success of current immunotherapies that block immune checkpoints, such as CTLA4 and PD1 in CD8 cells, has spurred interest in targeting other immune populations, such as NK cells. Given that blocking NK inhibitory receptors has proven beneficial in experimental tumor models, a number of blocking antibodies have been developed, with several progressing to clinical trials. Currently, KIR2D and NKG2A are the only two MHC-I-dependent receptors targeted to mimic a “missing-self” situation and enhance anti-tumor activity, with two agents that block KIR2D (Lirilumab and IPH2101) in clinical phase I or II trials. Lirilumab alone or in combination with other agents is being trialed against solid tumors (NCT03203876), hematological malignancies [154], chronic lymphocytic leukemia (NCT02557516), multiple myeloma (NCT01592370), resectable squamous cell carcinoma of the head and neck (NCT03341936), and resectable bladder cancer (NCT03532451), while IPH2101 is being trialed against acute myeloid leukemia [155]. Monalizumab is the only antibody being trialed for NKG2A, both alone and in combination with other agents for advanced gynecological solid tumors [156], advanced squamous cell carcinoma of the head and neck (NCT02643550), and resectable non-small cell lung cancer (NCT03794544).

In addition to KIR2D and NKG2A, anti-TIGIT antibodies are in multiple phase I and II clinical trials against different advanced solid tumors (NCT029133133, NCT02794571 and NCT02964013). Anti-TIGIT antibodies are also being used in combination with other therapies against solid tumors (NCT04150965, NCT03119428 and NCT04047862), notably against advanced non-small cell lung cancer (NCT03563716). These are just a handful of the clinical trials that are taking advantage of blocking inhibitory NK signaling, with no doubt many more to come.

## 4. Inhibitory NK Signaling

The full complement of events that transduce inhibitory receptor signals remains unclear, with much work still required to fully understand the different pathways. However, for some receptors a clearer picture of the signaling events following ligand engagement is now emerging, as well as identification of alternative signaling cascades that can lead to inhibition.

**Figure 3:**
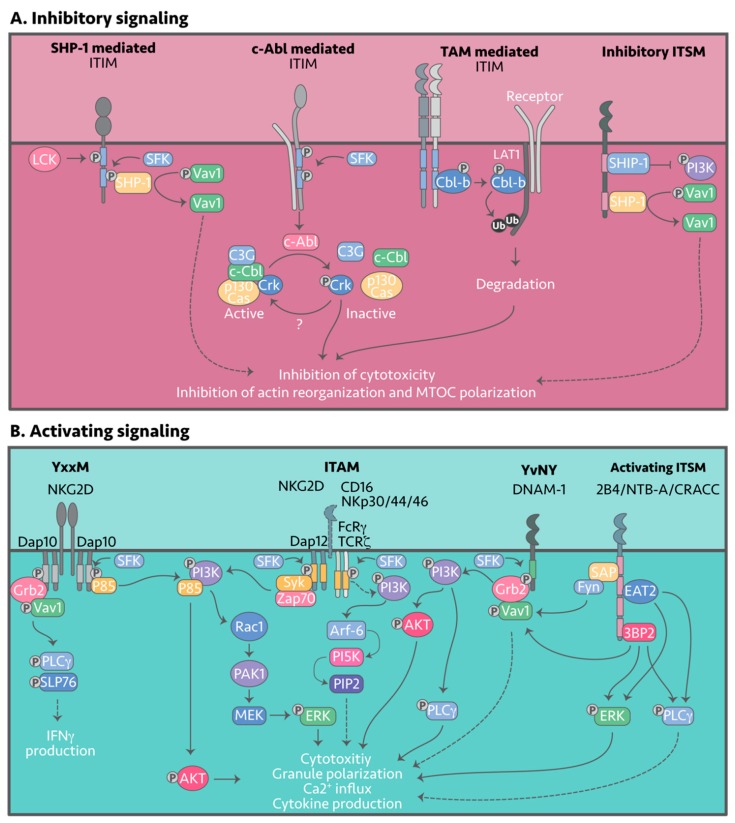
Inhibitory and activating NK cell signaling. NK cell effector function is collectively determined by the strongest activating or inhibitory signals. (**A**) Signaling downstream of inhibitory receptors is initiated by ligand engagement, followed by tyrosine phosphorylation of the signaling motif by Src-family kinases (SFK), Fyn or Lck. Once phosphorylated, there are three known inhibitory pathways: (1) Recruitment of SHP-1, SHP-2 or SHIP, which dephosphorylate Vav1; (2) Association with c-Abl kinase, which phosphorylates Crk disassociating it from its active complex and; (3) Phosphorylation and activation of Cbl-b by the TAM receptors. Cbl-b in turn ubiquitylates activating signaling intermediates such as LAT1 for degradation. (**B**) Signaling downstream of activating receptors is similarly transduced by tyrosine containing motifs that are phosphorylated by SFK. Various signaling intermediates such as Grb2, VAV1, or PI3K are then recruited, which induce cytotoxicity and cytokine release.

Inhibitory NK receptors signal via the different tyrosine-containing motifs: ITIM, ITT, ITSM, ITIM-like, ITT-like, or ITSM-like motifs. Upon engagement of the respective ligands, tyrosines within the signaling motifs are phosphorylated by members of the Src-family kinases (SFKs), with Lyn and Lck the most likely candidates in NK cells (Figure 3A) [157,158,159]. Tyrosine phosphorylation within the inhibitory motif enables recruitment of Src homology 2 (SH2)-containing protein tyrosine phosphatases such as SHP-1 and SHP-2, which then function to de-phosphorylate signaling intermediates and negatively regulate NK cell activity.

### 4.1. SHP1 and SHP-2 Signaling

SHP-1 is required for inhibition of NK cell effector function, whereas SHP-2, despite binding to most inhibitory receptors, appears to selectively inhibit cytokine production [160,161]. Most inhibitory NK receptor signaling tails are comprised of at least of two ITIMs or are found as homodimers creating two parallel ITIM sites within one receptor complex. Recruitment of SHP-1 and SHP-2 is dependent on both the residues flanking the tyrosine and the spacing between phosphorylated tyrosines. So far, the only known NK cell substrate de-phosphorylated by SHP-1 is the guanine nucleotide exchange factor Vav1. However, in other cell types SHP-1 has been shown to interact with CD3ζ, Syk, ZAP-70, LAT and SLP-76, and these may also prove to be targets in NK cells [159,162,163,164]. Phosphorylated Vav1 (pVav1) is a major intermediate downstream of multiple activating signals and is crucial for MTOC organization, thus dephosphorylation of pVav1 at the IS essentially attenuates NK activation. Signaling intermediates downstream of Src-kinase activity and SHP-1 and -2 recruitment remain to be elucidated in most KIRs, most Ly49s, CD94:NKG2A and B, NKRP1B, and CEACAM1 [165,166,167], whereas others such as KIR2DL1,3–4, Ly49C and gp49B1 bind to β-arrestin 2, enhancing recruitment of SHP-1 and SHP-2 [168]. For some receptors, recruitment of SHP-1 and SHP-2 is species dependent; take for instance human LAIR-1 associating with SHP-1 and SHP-2 and murine LAIR-1 only interacting with SHP-2 [145,169,170]. Regardless, the Src-kinase inhibitor PPI is shown to inhibit LAIR-1 tyrosine phosphorylation despite the Src-kinase responsible remaining unknown. Although LILRB1 contains four ITIM motifs, a study by Bellón et al. [171] showed that only Y614 and Y644 are required for inhibitory signals via SHP-1 association; Siglec-7 and -9 have also been shown to associate with SHP-1 and SHP-2 [172,173]. Inhibitory signaling by Siglec 7 is regulated by Suppressor of Cytokine Signaling (SOCS)3, which binds to the ITIM, blocking docking of SHP-1 and SHP-2, and potentially targeting Siglec-7 for proteasomal degradation via its associated E3 ubiquitin ligase complex [174]. In contrast, TIGIT contains an ITT-like motif that allows association with Grb2, which in turn recruits SHIP-1 (SH-2 containing inositol 5’ polyphosphatase 1) [175]. SHIP-1 is known to inhibit PI3K signaling by hydrolyzing PI(3,4,5)P3, inhibiting the accumulation and activation of Akt, Btk and PLC-γ (Figure 2A).

### 4.2. c-Abl Signaling

In both human and murine NK cells, a SHP-1/2-independent inhibitory mechanism has also been described, involving phosphorylation of a small adaptor protein named Crk by the c-Abl tyrosine kinase [176,177]. Active Crk is non-phosphorylated, is found in many different complexes together with c-Cbl, C3G and p130CAS, and is known to confer activating signals. Following engagement of inhibitory receptors and phosphorylation of the signaling motifs, the c-Abl kinase phosphorylates Crk, rendering it inactive and resulting in its dissociation [178] from activating complexes [179]. This inhibitory pathway has been shown to be KIR- and CD94:NKG2A-mediated.

### 4.3. TAM Signaling

A novel TAM/Cbl-b-mediated inhibitory signaling pathway has also been described. TAMs exert their inhibitory function by phosphorylation of human Cbl-b on tyrosine residues 133 and 363 [153]. Phosphorylation of Y363 prompts a conformational change, activating Cbl-b E3 ubiquitin ligase activity [180]. Cbl-b is thought to then target LAT1, a key molecule downstream of activating receptors NKG2D and NK1.1, for ubiquitination and subsequent proteasomal degradation, resulting in inhibition of NK cell activity [153].

## 5. Activating NK Cell Receptors

NK cells express activating receptors on their surface which recognize different stress molecules and ligands on ‘unhealthy’ cells (Figure 2B). Unlike the inhibitory receptors, most activating receptors lack a cytoplasmic signaling tail and instead associate with membrane bound adaptor proteins which contain immunoreceptor tyrosine-based activation motifs (ITAMs), to propagate their signals [181]. To date, CD16, NKG2D, NKp46 (natural cytotoxicity receptor 1; NCR1), DNAM-1, 2B4, NTB-A and CRACC have been found to be critical for tumor surveillance in both human and murine NK cells [182]. Human NK cells additionally express and rely on NKp30 (NCR3) and NKp44 (NCR2) for tumor surveillance (Figure 2 and Table 4) [183].

CD16 (FcgRIII) is a low-affinity receptor for IgG and is expressed at high levels on human NK cells and at lower densities on murine NK cells [194,195,196]. NK cells express a transmembrane form of CD16 that lacks a cytoplasmic signaling tail [197] and, in humans, associates with either CD3ζ homodimers [194,198], FCRγ homodimers [199] or CD3ζ:FCRγ heterodimers to transduce activating signals, while murine CD16 associates with FCRγ homodimers [200]. CD16 expression enables NK cells and other immune cells to recognize the Fc portion of an antibody bound to an antigen on the surface of tumor cells and trigger NK-mediated lysis of the target cell (known as ADCC). After cytolysis, CD16 is proteolytically cleaved by ADAM17 [201] or MMP25 [202] and shed from the NK cell surface, with shedding not only important for NK cell detachment and subsequent targeting of tumor cells but also enhancement of subsequent NK cell signaling [203]. This has resulted in a new field focused on generating monoclonal antibodies against tumor antigens to enhance immune-mediated ADCC [204]. Additionally, endogenous antibodies to tumor antigens have been observed in patients with various cancers, including human papillary thyroid cancer, some soft tissue sarcomas and melanoma [205,206]. Although these antibodies are predominantly IgG and are located within neoplastic tissue in the TME [207], they apparently fail to elicit tumor control; why this is the case remains unknown.

NKG2D is a member of the NKG2 receptor family and is one of the main activating receptors involved in NK cell tumor surveillance [208]. In humans, its ligands include MICA and B and UL16-binding proteins (ULBP)1–6, and in mice, Rae1α-ε, MULT1 and H60a-c (with H60a-c restricted to BALB/C mice) [188,189,190]. Structurally, NKG2D ligands resemble MHC-I proteins, with MICA and B containing three extracellular α(1–3) domains in which α3 is an Ig-like domain. ULBP1-6 have two extracellular α(1–2) domains and ULBP4 and 5 are similar to murine Rae1 ligands [191,209]. ULBP1-3 and -6 are attached to the membrane via a glycophosphatidylinositol (GPI)-anchor, whereas MICA and B and ULBP4–5 all have a transmembrane domain and cytoplasmic tail.

NKG2D has two isoforms that dictate NKG2D signaling: the short isoform NKG2D-S associates with the DNAX-activating protein (DAP)10 and 12 adaptor proteins, while the long isoform (NKG2D-L) only associates with DAP10 [210]. Homodimeric NKG2D forms a hexamer with each subunit associated with either a DAP10 or DAP12 homodimer, respectively [211]. Additionally, NKG2D-S is restricted to mice, meaning that human NKG2D only signals via DAP10, while engagement of the murine NKG2D receptor can lead to signaling by either DAP10 or DAP12 [212,213]. The differences between mouse and human are further amplified by the presence of an ITAM signaling motif in DAP12 and a YxxM motif in DAP10 (Figure 2B and Table 5) [214,215].

Human NKG2D ligands are expressed in ovarian cancer, leukemia, colorectal cancer and pediatric brain cancers, amongst others [216,217,218,219,220]. Regardless of ligand expression, Groh et al. [221] showed that soluble MIC (sMIC) shed by tumors impaired NKG2D mediated cytotoxicity by blocking receptor engagement and thus signaling [221,222]. This shedding of NKG2D ligands has been observed in many cancers, including ovarian, breast, lung, colon and prostate, as well as lymphoma, myeloma and melanoma [219,220,223,224]. Antibodies targeting sMIC have been shown to enhance NK and T cell-mediated killing of head and neck squamous cell carcinoma [225], in addition to other patient cancers, when combined with PD1/PD-L1 blockade [226].

NKp46, NKp30 and NKp44 (NCR family) were the first activating receptors identified in NK cells [227,228,229]. While many molecules such as heparin, vimentin and viral proteins are suspected ligands, the search for cellular ligands remains a priority in the field [183]. So far, two cellular ligands, B7 homologue 6 (B7H6) and NKp44L, have been identified for NKp30 and NK44, respectively [184,185,186,187]. There is currently no known ligand for NKp46, which is the only NCR conserved in both human and mice and is found on activated and resting NK cells. Despite not knowing which ligands activate NKp46, its engagement has been shown to be critical for control of melanoma and lung carcinoma metastasis in experimental models [230,231]. NKp46 contains two extracellular Ig-like domains, a transmembrane domain and a short, 25-residue cytoplasmic tail [232]. Following NKp46 engagement, signaling occurs through NKp46 interaction with a CD3ζ:FCRγ heterodimer [229,233] and results in NK cytoskeletal engagement and F-actin accumulation at the lytic synapse, both early events in the killing process (Figure 1) [234]. Tantalizingly, this hints that NKp46 has a critical role in target identification, but without a known ligand or ligands, it is impossible to confirm. In addition, studies have shown that NKp46 engagement results in IFNγ and TNFα production [231,235].

NKp30 is found on mature resting and activated NK cells, with most NK cells expressing one or two of the three major NKp30 isoforms (NKp30A-C), that differ in the composition of their cytoplasmic tails [228,236]. Depending on the isoform engaged, NKp30 transduces different signals via CD3ζ homodimers or CD3ζ:FCRγ heterodimers. For example, engaging NKp30A or B induces significantly higher IFNγ, TNFα and IL12B production, while NKp30C which induces IL10 production, leads to immune suppression [32,237]. In addition, NKp30B constitutively associates with CD3ζ, while both NKp30A and C associate with CD3ζ upon engagement; however, NKp30A has a tighter association with CD3ζ and gives the dominant response [32]. Furthermore, the predominant expression of NKp30C in patients with GIST is predictive of a poor prognosis [32,237].

NKp44 is only expressed upon activation and associates with ITAM-containing DAP12 to induce IFNγ and TNFα production [229,238,239], and cell lysis of experimental cervix carcinoma and neuroblastoma cell lines [240]. Finally, although NKp44 contains a cytoplasmic ITIM-like motif, this does not appear to be functional [239,241].

DNAM-1 has an important role in NK cell-mediated tumor immunosurveillance and shares CD155 and CD112 ligands with TIGIT and TACTILE (CD96) [242,243]. The role of DNAM-1 in immunosurveillance was discovered using the RMA lymphoma model where NK cells were shown to be responsible, at least in part, for metastatic control of DNAM-1 ligand-expressing RMA tumors [244]. The importance of DNAM-1 is underscored by DNAM-1-deficient NK cells that no longer control experimental metastasis in a spontaneous fibrosarcoma formation model [245,246]. However, if the tumors express NKG2D ligands, then DNAM-1 deficiency has no impact, suggesting a signal hierarchy between the two receptors [247,248]. DNAM-1 ligand CD155 is expressed in a variety of human cancers, including colon, adenocarcinoma, pancreatic and melanoma [249,250,251,252,253]. Interestingly, human melanoma samples have also been shown to express CD112 [254], and expression of CD112 and CD155 on patient-derived neuroblastomas correlated with susceptibility to NK cell killing in vitro [255]. To date, CD155 but not CD112 has been shown to be critical for DNAM-1-mediated NK cell cytotoxicity [255,256]. Furthermore, CD155 association with DNAM-1 promotes NK cell cytotoxicity and IFNγ production [257].

2B4 (SLAMF4), NTB-A (SLAMF6 or Ly108 in mouse) and CRACC (SLAMF7) belong to the signaling lymphocytic activation molecule (SLAM) family and are the only members expressed on NK cells [258,259]. CRACC and NTB-A act in *trans* as their own ligands, while CD48 (SLAMF2) is the ligand for 2B4 and is thought to act in trans and in cis [192,193,260]. CRACC and 2B4 are potent stimulators of NK cell cytotoxicity; CRACC is already in clinical use and 2B4 is a potential new therapeutic target [261]. The SLAMs contain cytoplasmic ITSM motifs that recruit different signaling molecules to allow for a switch between activating and inhibitory signals following receptor engagement [262,263].

## 6. Current Therapies Harnessing the Power of Activating NK Receptors

There are several ongoing clinical trials testing antibodies that enhance NK cell activation, mediate direct cell killing (ADCC) or achieve both NK cell activation and ADCC. The latter is exemplified by Elozutumab, an anti-CRACC (SLAM7) antibody currently in pre-clinical testing and phase 1–3 clinical trials for multiple myeloma (NCT01335399) [264,265,266]. Another ongoing trial in non-Hodgkin’s lymphoma is combining anti-CD123 antibody with adoptive transfer of an NK cell line engineered to express high levels of CD16 and potentiate NK responses (NCT03027128) [267]. Adoptively transferred, allogeneic CD19 CAR-NK cells were successfully used in recent phase 1 and 2 trials to treat patients with non-Hodgkin’s lymphoma or chronic lymphocytic leukemia (CLL) without significant toxicities [35]. These studies demonstrate the importance of NK cell therapies and pave the way for further clinical trials using blocking antibodies and/or CAR-NK cells expressing activating receptors [268,269,270].

## 7. Activating NK Signaling

### 7.1. ITAM Signaling

Following CD16, NKG2D and NCR family receptor engagement adaptor proteins, DAP12, CD3ζ and FCRγ are rapidly phosphorylated within their ITAM sequences by an as yet unidentified Src-kinase, which leads to adaptor association with Syk or Zap70 tyrosine kinases (Figure 3B) [215,271,272]. Following recruitment to DAP12, Syk is thought to interact with the p58 subunit of PI3K leading to a PI3K → Rac1 → PAK1 → MEK → ERK signaling cascade that drives NK cell cytotoxicity (Figure 3B) [272,273]. Although Zap70 has also been shown to associate with the ITAMs it does not appear to be required for signaling.

CD16 signals through its CD3ζ or FCRγ adaptors and like DAP12, activates PI3K, however, other signaling molecules such as Vav1, PLC-γ1 and PLC-γ2 can also be activated following CD16 engagement [274,275]. Additionally, CD16 engagement has been linked to PIP2 production mediated by PI5K [276], with Galandrini et al. [277] showing that PI5K was required for NK cell degranulation but not granule polarization in primary human NK cells. The combined activation of the PI3K and PI5K pathways could explain why CD16 is the only receptor that can fully activate resting human NK cells [278]. In addition to the ITAM-mediated signaling cascades, NK cells have been shown to signal through transmembrane-bound LAT complexed with PLC-γ1/2; the signaling intermediates remain to be elucidated [279].

### 7.2. DAP10 (YxxM) Signaling

DAP10 is a small transmembrane adaptor protein containing a ’traditional’ costimulatory PI3K binding motif (YxNM) and a binding site for Grb2 (pYxNx) [280]. Following receptor engagement, the DAP10 motif is phosphorylated by an unknown Src-kinase to recruit a Grb2-Vav1 complex and the p85 subunit of PI3K [281,282]. Phosphorylation of Grb2-Vav1 leads to phosphorylation of Vav1, PLC-γ2 and SLP-76 [281,283]. Presumably, PI3K activation via DAP10 converges on AKT with the end result being an increase in direct cytotoxicity [280,284]. Interestingly, Grb2-Vav1 signaling alone is not sufficient to stimulate full calcium release and cytotoxicity [282], whilst NKG2D:DAP10 activation of Vav1 is important for induction of actin polymerization and polarization of MTOC at the IS [285].

### 7.3. DNAM-1, 2B4, CRACC and NTB-A Signaling

DNAM-1, 2B4, CRACC and NTB-A contain a cytoplasmic signaling tail, distinguishing them from the NCRs, CD16 and NKG2D.

DNAM-1 has an ITT-like motif that is phosphorylated at Y319 in mouse and Y322 in humans [286] and is required for association with Grb2 and initiation of the PI3K signaling cascade (Grb2→ Vav1 → PI3K → PLC-γ1) (Figure 3B) [102], although further signaling intermediates have not been fully elucidated. Interestingly, DNAM-1 signaling enhances Vav1-mediated actin polymerization and polarization of the lytic granules to the IS, consistent with its role in NKG2D:Dap10 signaling [102,285].

2B4, CRACC and NTB-A: 2B4 contains four ITSM motifs, while CRACC and NTB-A contain two ITSM motifs [287]. Following 2B4 engagement, ITSM tyrosines are phosphorylated recruiting either SAP, EAT2 or 3BP2. Six possible signaling cascades have been elucidated to date: (1) SAP → Fyn → pVAV1 [288,289], (2) EAT → PLC-γ → Ca2+ flux, (3) EAT → pERK, [290], (4) 3BP2 → pVAV1, (5) 3BP2 → PLC-γ and (6) 3BP2 → pERK [291,292,293]. Notably, Saborit-Villarroya et al. [292] showed that cytotoxicity, not cytokine release, is regulated by pVAV1 and ERK, thus cytokine release and cytotoxicity are regulated by different signaling pathways downstream of 2B4.

Similar to 2B4, engagement of NTB-A results in recruitment of SAP and EAT2, and NK cell cytotoxicity and cytokine production [258,294,295]. In contrast, CRACC does not recruit SAP and solely relies on EAT2 for signaling [296,297]. As mentioned, ITSM phosphorylation can transduce either activating or inhibitory signals (Figure 2A). Activation relies on SAP recruitment, which blocks binding site of the phosphatases SHP-1, SHP-2 and SHIP [288,298,299]. Patients suffering from X-linked lymphoproliferative disease (XLP), which is caused by mutations in the gene encoding SAP, show defective NK cell activation and even inhibition [300,301].

## 8. Releasing the Brake

While the previous sections discuss the inhibitory and activating receptors and their signaling events separately, in reality, inhibitory and activating signaling occurs simultaneously upon each NK cell-to-cell encounter. As mentioned, NK cells are loaded with lytic granules and are ready to kill once a “decision” has been made. It is this ability to release lytic content and kill their targets within 30 minutes of engagement, that makes them such a powerful weapon for the immune system [302,303,304]. These natural killers are constantly surveilling our bodies and more often than not, encounter healthy cells that engage the myriad of inhibitory receptors expressed on NK cells. NK cells can thus be considered in a constant state of inhibition and overcoming that inhibitory threshold requires either “release” of the inhibitory “brake” or a countermanding activating signal such as via CD16, or from co-engagement of various activating receptors [305]. The inhibitory signals appear to act by blocking early activation, for example, NKG2D, DNAM-1, 2B4, NTB-A and CRACC all converge at Vav-1 dephosphorylation (Figure 3A), which would prevent MTOC formation and result in tolerance. This implies that Vav phosphorylation may be a “master” switch and understanding the regulatory events surrounding it could point to new interventions which release the brake on NK cell killing.

## 9. Conclusion and Future Directions

While numerous inhibitory and activating receptors have been identified, the NK signaling field is still in its infancy. For clarity, this review has focused on the most well-studied aspects and we apologize if we have omitted publications that have contributed to a rapidly moving field.

NK cells play a pivotal role in controlling tumor metastasis and there is enormous potential for the development of new cancer immunotherapies that enhance NK cell activity and infiltration into the TME. If we consider that NK cells are constantly surveying the body for infected or transformed “unhealthy” cells, then essentially, most of the time there should be no NK response and thus constant NK inhibitory signaling in the “normal” state.

In this context, the trigger for response is activation and raises an over-arching question: how does activation signaling switch off or dominate baseline inhibitory signaling? There are still many gaps in our understanding of these activating and inhibitory signaling pathways. For instance, what are the initiating signals post-ligand–receptor engagement? What is the role of the Src-family kinases and how are they activated? Similarly, once the initial signals are transduced, what intermediates and regulatory mechanisms come into play and how does this converge to give the final killing blow? Further delineation of the signals that regulate NK cell responses will be critical not only in the identification of new targets but to understand the full impact of any intervening strategies.

To evade NK cell recognition and killing, tumor cells upregulate inhibitory ligands and signaling, as well as downregulate activating ligands; blocking inhibitory and/or enhancing activating ligands offers attractive opportunities to improve anti-tumor responses. In contrast to T-cells, activated or adoptively transferred NK cells have the added advantage of not inducing cytokine release syndrome or neurotoxicity, and allogeneic NK cells/cell lines have at least proven safe for adoptive immunotherapy [35,306,307]. Further safety and efficacy testing of NK cell therapies, such as adoptive transfer of NK cell lines, CAR-NK cells, checkpoint blockades and ADCC, will no doubt further advance the field of NK therapeutics.

Many studies have shown that NK cell–mediated tumor control results not only from NK cell cytotoxicity but also NK release of cytokines into the TME to marshal other immune cells into a full-scale attack. Therapeutic approaches that not only trigger cytotoxicity, but aid exhaustion recovery and enhance proliferation and directed cytokine production, will be key.

## Figures and Tables

**Table 1:** Consensus sequence of signaling motifs.

Signaling Motifs	Consensus Sequence
ITIM	S/I/V/LxYxxI/V/L
ITT/ITT-Like	YxNM/YvNy
ITSM	TxYxxV/I
ITAM	YxxI/Lx6-12YxxI/L

**Table 2:** NK cell inhibitory receptors and their ligands [56,57,58].

**Human Receptor**	**Classical MHC Ligands**
KIR2DL1	HLA-C group 2 molecules (Asn77 and Lys80) [45]
KIR2DL2	HLA-B and HLA-C group 1 molecules (Ser77 and Asn80) [45]
KIR2DL3	HLA-C group 1 molecules (Ser77 and Asn80) [45] and some HLA-CGroup 2 and HLA-B (weaker affinity than 2DL2) [46]
KIR2DL5(A+B)	unknown
KIR3DL1	HLA-A and B with the Bw4 epitope, amino acid positions 77–83 [45]
KIR3DL2	Some HLA-A allotypes [45]
KIR3DL3	Unknown
LILRB1	HLA-C [48,49]
**Human receptor**	**Non-classical MHC ligands**
CD94/NKG2A/B	HLA-E [59,60]
LILRB1	HLA-E and HLA-G [61] and UL18 (MHC viral homologue) [62,63]
**Human receptor**	**Non-MHC-I ligands**
SIGLEG-7/9	α2,3- and α2,6-linked sialylated proteins [64,65,66]
NKRP1A	LLT1 (CLEC2D) [67,68]
**Mouse**	**Classical MHC ligands**
Ly49A	H2-D^d^ [11,69,70,71,72,73,74], H2-D^k^ [74], H2-L^d^, H2-K^b^, H2-K^b^ and H2-D^p^ [69]
Ly49B	Unknown
Ly49C	H2-K^b^ [74,75], H2-K^d^ [74], H2-K^k^, H2-D^b^, H2-D^d^ [76]
Ly49F	H2-D^d^ [76]
Ly49G	H2-D^b^ [69]
Ly49I	H2-K^b^ [75] and H2-K^d^ [74]
**Human receptor**	**Non-classical MHC ligands**
Ly49A	H2-M3 [77]
Ly49C	H2-Q10 [78]
CD94/NKG2A/B	Qa-1 [59,60]
**Human receptor**	**Non-MHC-I ligands**
NKRP1B	Clr-b (Clec2d) [79]
gp49B1	integrin αVβ3 [80]
Ly49E	urokinase plasminogen activator (uPA) [81]
**Shared receptors**	**Non-MHC-I ligands**
TIGIT	poliovirus receptor (CD155, PVR) and nectin-2 (CD112, PVRL2) [82,83]
TACTILE	poliovirus receptor (CD155, PVR) and nectin-2 (CD112, PVRL2) [82,83]
CEACAM1	CEACAM1 [84]
LAIR-1	Collagen [85]
KLRG1	E- and N-Cadherin [86,87]
TAMs	Gas6 and Pros1 [88,89]

**Table 3:** Inhibitory NK cell receptors and their signaling motifs.

**Human**	**Form**	**Signaling Motif ^1^**		**pY ^2^ Position**	**Reference**
KIR2DL1	Monomer	2× ITIM	VTYTQL	Y302	[44]
			IVYTEL	Y332	
KIR2DL2	Monomer	2× ITIM	VTYTQL	Y302	[44]
			IVYAEL	Y332	
KIR2DL3	Monomer	2× ITIM	VTYAQL	Y303	[44]
			IVYTEL	Y333	
KIR2DL5	Monomer	2× ITIM	VTYAQL	Y298	[44]
			TMYMEL	Y228	
KIR3DL1	Monomer	2× ITIM	VTYAQL	Y398	[44]
			ILYTEL	Y428	
KIR3DL2	Monomer	2× ITIM	VTYAQL	Y398	[44]
			SVYTEL	Y428	
KIR3DL3	Homodimer	1× ITIM	VTYAQL	Y381	[44,139]
LILRB1/ILT2	Monomer	4× ITIM	NLYAAV	Y533	[50,51,140]
			VTYAEV	Y562	
			VTYAQL	Y614	
			SIYATL	Y644	
SIGLEC-7/9	Monomer	1× ITIM	IQYAPL/LQYASL	Y437/Y433	[131,132]
		1× ITIM	NEYSEI/TEYSEI	Y460/Y456	
NKRP1A	Homodimer	1× ITIM-like	AIYAEL	Y7	[141]
**Mouse**					
Ly49A	Homodimer	1× ITIM	VTYSMV	Y8	[93,142]
Ly49B	Homodimer	1× ITIM	VTYTTL	Y8	[93,142]
Ly49C, E-I	Homodimer	1× ITIM	VTYSTL	Y8	[93,142]
NKRP1B	Homodimer	ITIM	LVYADL	Y8	[141]
**Shared**					
iNKG2A/B	Heterodimer with CD94	2× ITIM	(h) VIYSDL	Y8	[95,96]
			(m) VTYAEL		
		1× ITIM	(h) EITYAEL	Y40	[95,96]
		1× ITIM-like	(m) IIYSDF		
TIGIT	Monomer	1× ITT-like	YFN	(h) Y225	[82,83]
				(m) Y230	
		1× ITIM	LSYRSL	(h) Y231	
				(m) Y236	
TACTILE	Monomer	1× ITIM	IKYTCI	(h) Y566	[105]
				(m) Y583	
		1× YxxM	(h) YHEM	(h) Y579	
CEACAM1	Homomer Homodimer Oligomer	(h) 2× ITIM	VTYSTL	Y459	[84,143,144]
			IIYSEV	Y486	
		(m) 1× ITIM	VAYTVL	Y454	
		(m) 1× ITSM	TVYSEV	Y481	
LAIR-1	Monomer	2× ITIM	(h) VTYAQL	Y251	[145]
			(h) ITYAAV	Y281	
			(m) VTYIQL	Y228	
			(m) STYAAI	Y257	
KLGR1	Homodimer	1× ITIM	(h) VIYSML	Y7	[146,147]
			(m) SIYSTL		
**Shared**	**Form**	**Signaling Motif ^1^**		**pY ^2^ Position**	**Reference**
Tyro	Homodimer Heterodimer	1× ITIM-like	IYNYL	(h) Y742 (m) Y744	[148]
Axl	Homodimer Heterodimer	1× ITIM	LLYSRL	(h) Y634 (m) Y628	[148]
		1× ITIM-like	IYDYL	(h) Y759/761 (m) Y753/755	
Mer	Homodimer Heterodimer	1× ITIM	LLYSRL	(h) Y685 (m) Y680	[148]
		1× ITIM-like	MYDYL	(h) Y810/Y812 (m) Y805/807	

^1^ m = mouse, h = human; ^2^ Phosphorylated tyrosine (pY).

**Table 4:** Activating NK receptors paired to cellular ligands and signaling adaptors.

Human	Ligands	Co-receptor
NKp30	B7H6 [184,185]	CD3ζ and FCRγ
NKp44	NKp44L (unusual isoform of MLL5) [186,187]	DAP12
**Shared**		
NKG2D	MICA/B, ULBP1-6 [188,189] (humans)	DAP10
	Rae1B, MULT1, H60 [190,191] (mice)	DAP10 and DAP12
NKp46	Unknown	CD3ζ and FCRγ
DNAM-1	CD155 and CD112 [82,83]	-
2B4	CD48 (SLAMF2) [192,193].	-
NTB-A	NTB-A (SLAMF6, CD319 and Ly108 in mice) [192,193].	-
CRACC	CRACC (SLAMF7, CD352) [192,193].	-
CD16	IgG [194,195]	CD3ζ and FCRγ

**Table 5:** Activating signaling motifs.

**Adaptor Proteins**	**Signaling Motif**	**Motif Sequence**	**pY ^1,2^ Position**
DAP10	YxxM	YINM	(h) Y86
			(m) Y71
DAP12	ITAM	YQELQGQRSDVYSDL	(h) Y91 & Y102
	ITAM	YQELQGQRPEVYSDL	(m) Y92 & Y103
FCR γ	ITAM	YTGLSTRNQETYETL	Y65 & Y76
CD3ζ	3x ITAM	YNELNLGRREEYDVL	Y72 & Y83
		YNALQKDKMAEAYSEI	Y111 & Y123
		YQGLSTATKDTYDAL	Y142 & Y153
**Receptor**	**Signaling Motif**	**Motif Sequence**	**pY^1^ Position**
NKp44	ITIM-like	EILYHTVA	(h) Y258
DNAM-1	ITT-like	YvNY	(h) Y322 & Y325
			(m) Y319 & Y322
2B4	ITSM 1	TIYEDV	(h) Y271
		TIYEYV	(m) Y266 & Y268
	ITSM 2	TIYSMI	(h) Y297
		TMYSMI	(m) Y325
	ITSM 3	TLYSLI	(h) Y317
		TVYSVV	(m) Y343
	ITSM 4	T IYEVI	(h) Y342
		TVYEEV	(m) Y369
NTB-A	ITSM 1	TVYASV	(h) Y285
		TVYAQV	(m) Y295
	ITSM 2	TIYSTI	(h) Y309
		TIYS IV	(m) Y319
CRACC	ITSM-like	TEYDTI	(h) Y284
		ADYDTI	(m) Y282
	ITSM	TVYSTV	(h) Y304
		TFYSTV	(m) Y302

^1^ m = mouse, h = human; ^2^ Phosphorylated tyrosine (pY).

## Abbreviations

Antibody dependent cellular cytotoxicity (ADCC); Programmed cell death protein 1 (PD1); Programmed death-ligand 1 (PD-L1); B7 homologue 6 (B7H6); Bruton’s tyrosine kinase (Btk); Abelson tyrosine kinase (c-Abl); c-Casitas B lineage lymphoma (c-Cbl); Chimeric antigen receptor (CAR); Casitas B lineage lymphoma b (Cbl-b); Carcinoembryonic Ag cell adhesion molecule 1 (CEACAM1); Carcinoembryonic Ag cell adhesion molecule 1 Ligand (CEACAM1-L); Chronic lymphocytic leukemia (CLL); CD2-like receptor-activating cytotoxic cell (CRACC); Cytotoxic T-lymphocyte-associated protein 4 (CTLA4); C-type lectin domain (CTLD); DNAX-activating Protein 10 (DAP10); DNAX-activating Protein 12 (DAP12); DNAX accessory molecule-1 (DNAM-1); Extracellular signal-regulated kinases (ERK); Filamentous actin (F-actin); Low affinity immunoglobulin gamma Fc region receptor III-A (FcgRIII); Growth arrest-specific gene 6 (Gas6); Gastrointestinal sarcoma (GIST); Growth factor receptor-bound protein 2 (Grb2); Human leukocyte antigens (HLA); Interferon gamma (IFNγ); Immunoglobulin (Ig); Inhibitory KIRs (iKIRs); Interleukin-12 subunit beta (IL12B); Inhibitory Ly49 receptors (iLy49s); Inhibitory NKG2 (iNKG2); Immunological synapse (IS); Immunoreceptor tyrosine-based activation motif (ITAM); Immunoreceptor tyrosine-based inhibtory motif (ITIM); Immunoreceptor tyrosine-based switch motif (ITSM); Immunoglobulin tail tyrosine motif (ITT); Killer cell immunoglobulin-like receptor (KIR); Killer cell lectin-like receptor subfamily A (klra); Killer cell lectin-like receptor G1 (KLRG1); Soluble leukocyte-associated Ig-like receptor-1 (LAIR-1); L-type amino acid transporter 1 (LAT1); lymphocyte-specific protein tyrosine kinase (Lck); Leukocyte immunoglobulin-like receptor (LILR); Lymphocyte Ag (Ly(X); Mitogen-activated protein kinase (MAPK); Major histocompatability complex (MHC); MHC class I-related chains (MIC); Matrix metalloproteinase 25 (MMP25); Microtubule-organizing center (MTOC); Murine UL16 binding protein (MULT); Natural cytotoxicity receptor (NCR); Natural killer (NK); Killer cell lectin-like receptor subfamily B, member 1 (NK1.1); Non-obese diabetic (NOD); NK-T-B-Antigen (NTB-A); Phosphatidylinositol 3-kinase (PI3K); Phosphatidylinositol 4,5-bisphosphate or PtdIns(4,5)P2 (PIP2); Phospholipase C (PLC-y); Proton-pump inhibitors (PPI); Protein S (Pros1); Phosphorylated Vav1 (pVav1); Poliovirus receptor (PVR); Poliovirus Receptor-Related 2 (PVRL2); Ras-related C3 botulinum toxin substrate 1 (Rac1); Retinoic Acid Early Transcript 1E (RAET1E); Receptor tyrosine kinases (RTKs); SLAM-associated protein (SAP); Src-family kinases (SFKs); Src homology 2 (SH2); SH2-containing inositol 5-phosphatase (SHIP); SH2 domain- containing protein tyrosine phosphatase-1 (SHP-1); SH2 domain- containing protein tyrosine phosphatase-2 (SHP-2); Sialic acid-binding immunoglobulin-like lectin (Siglec); Signaling lymphocytic activation molecule (SLAM); Lymphocyte cytosolic protein 2 (SLP-76); Soluble MIC (sMIC); Suppressor of Cytokine Signaling (SOCS); Proto-oncogene tyrosine-protein kinase Src (Src); Spleen tyrosine kinase (Syk); Tyro3, Axl and MerTK (TAM); T cell immunoglobulin and ITIM domain (TIGIT); Tumor microenvironment (TME); Tumor necrosis factor alpha (TNFα); Tumor necrosis factor-related apoptosis-inducing ligand (TRAIL); UL16-binding proteins (ULBP); X-linked lymphoproliferative disease (XLP); Zeta Chain Of T Cell Receptor Associated Protein Kinase 70 (ZAP-70).
